# Simultaneous detection of four different neuraminidase types of avian influenza A H5 viruses by multiplex reverse transcription PCR using a GeXP analyser

**DOI:** 10.1111/irv.12370

**Published:** 2016-02-09

**Authors:** Meng Li, Zhixun Xie, Zhiqin Xie, Jiabo Liu, Liji Xie, Xianwen Deng, Sisi Luo, Qing Fan, Li Huang, Jiaoling Huang, Yanfang Zhang, Tingting Zeng, Jiaxun Feng

**Affiliations:** ^1^ Guangxi Key Laboratory of Animal Vaccines and DiagnosticsGuangxi Veterinary Research InstituteNanningGuangxiChina; ^2^ Guangxi Key Laboratory of Subtropical Bioresources Conservation and UtilizationCollege of Life Science and TechnologyGuangxi UniversityNanningGuangxiChina

**Keywords:** Differential diagnoses, GeXP analyser, H5 avian influenza viruses, HA typing, multiplex detection, NA typing

## Abstract

**Objectives:**

In order to develop a multiplex RT‐PCR assay using the GeXP analyser for the simultaneous detection of four different NA serotypes of H5‐subtype AIVs, effective to control and reduce H5 subtype of avian influenza outbreak.

**Design:**

Six pairs of primers were designed using conserved and specific sequences of the AIV subtypes H5, N1, N2, N6 and N8 in GenBank. Each gene‐specific primer was fused at the 5′ end to a universal sequence to generate six pairs of chimeric primers, and one pair of universal primers was used for RT‐PCR, and PCR product separation and detection were performed by capillary electrophoresis using the GenomeLab GeXP genetic analysis system.

**Setting:**

Single and mixed avian pathogen cDNA/DNA templates were employed to evaluate the specificity of a multiplex assay with a GeXP analyser. Corresponding specific DNA products were amplified for each gene, revealing amplification peaks for M, H5, N1, N2, N6 and N8 genes from four different NA subtypes of influenza A H5 virus.

**Sample:**

A total of 180 cloacal swabs were collected from poultry at live bird markets.

**Main outcome measures:**

The multiplex PCR assay demonstrated excellent specificity, with each pair of specific primers generating only products corresponding to the target genes and without cross‐amplification with other NA‐subtype influenza viruses or other avian pathogens. Using various premixed ssRNAs containing known AIV target genes, the detection limit for the multiplex assay was determined to be 10^2^ copies/μl. The GeXP assay was further evaluated using 180 clinical specimens and compared with RRT‐PCR (real‐time reverse transcriptase PCR) and virus isolation.

**Conclusions:**

This GeXP analyser‐based multiplex assay for four different NA subtypes of H5 HPAI viruses is both highly specific and sensitive and can be used as a rapid and direct diagnostic assay for testing clinical samples.

## Introduction

Influenza virus serotypes are classified based on the combination of two major antigens on the virion, namely haemagglutinin (HA) and neuraminidase (NA). To date, 16 H types and 9 N types have been acknowledged, and a 17th and 18th HA type plus a 10th and 11th NA type have recently been discovered in bats.^1^, ^2^ Avian influenza is an acute infectious disease caused by type A avian influenza viruses (AIVs), and certain subtypes have public health significance because they can also infect humans. In addition to causing considerable economic damage to the global poultry industry, highly pathogenic avian influenza viruses (HPAIVs) pose a major public health hazard, especially the H5 subtype.^3^ Since 2003, H5N1 HPAI viruses have emerged in at least sixty countries worldwide,^4^, ^5^ and various NA subtypes of H5 HPAIVs (H5N2, H5N6 and H5N8) have recently been detected in different poultry and wild birds, with highly pathogenic AI epidemics. For example, Heilongjiang, Jiangsu and Hunan provinces, as well as other provinces in China, reported outbreaks of H5N6 on poultry farms in 2014 and 2015; in 2014, the People's Republic of China, Japan, Germany, the Netherlands, the UK and the Republic of Korea reported outbreaks of H5N8 on poultry farms and also viral presence in migratory birds. Furthermore, earlier this year, Taiwan reported outbreaks of H5N8 and H5N2 on poultry farms, and H5N2 HPAI viruses appeared in at least 14 states in the USA.^6^ These AIV outbreaks resulted in extensive losses to the poultry industry worldwide.^7^

Laboratory diagnosis is essential when AI is suspected in the field, and currently, RT‐PCR tests are used routinely for AI diagnosis and genotyping. Although conventional and real‐time RT‐PCR protocols have been published for the detection of H5‐subtype HPAIVs in poultry,^8^, ^9^, ^10^ none of these methods can be applied for simultaneous detection or for HA and NA subtyping of H5‐subtype HPAI viruses such as H5N1, H5N2, H5N6 and H5N8 in a single assay. The GeXP analyser is a multiplex gene expression profiling analysis platform developed by Beckman Coulter Company (Brea, CA, USA) that was originally designed to allow for the high‐throughput, robust and differential assessment of a multiplexed expression profile of up to 35 genes in a single RT‐PCR.^11^ This system has been successfully used for the rapid identification of several viral diseases of humans, such as nine serotypes of enteroviruses associated with hand, foot and mouth disease,^12^ pandemic influenza A H1N1 virus,^13^ sixteen human respiratory virus types/subtypes,^14^ and seven enteric viruses associated with acute gastroenteritis,^15^ as well as the genotyping of 11 human papillomaviruses.^16^ Moreover, our laboratory has successfully developed three GeXP multiplex PCR assay for the simultaneous typing of nine different avian respiratory pathogens, eight swine reproductive and respiratory pathogens, as well as differentiation of eleven duck viruses.^17^, ^18^, ^19^ In this study, a multiplex RT‐PCR assay using the GeXP analyser was developed for the simultaneous detection of four different NA serotypes of H5‐subtype AIVs.

## Methods

### Virus strains and DNA/RNA extraction

The AIV reference strains, field isolates and other avian pathogens used in this study are listed in Table 1. Genomic DNA/RNA was extracted from 200 μl of virus using MiniBEST Viral RNA/DNA Extraction Kit ver. 5.0 (TaKaRa, Dalian, China) according to the manufacturer's protocol. DNA and RNA were eluted in 30 μl of nuclease‐free water. The concentrations of total DNA and RNA were measured by UV spectrophotometry (Beckman Coulter). All DNA and RNA samples were stored immediately at −80°C until used in experiments.

**Table 1:** Sources of pathogens used and GeXP assay results

Pathogens/field samples	Numbers of sample	Source	Results					
			M	H5	N1	N2	N6	N8
Reference samples								
Inactivated H5N1 AIV Re‐1	1	HVRI	+	+	+		−	−
cDNA of H5N3 AIV Duck/HK/313/78	1	CU	+	+	−	−	−	−
cDNA of AIV H5N2/chicken/QT35/87	1	CU	+	+	−	+	−	−
cDNA of AIV H5N5/chicken/QT35/98	1	CU	+	+	−	−	−	−
cDNA of AIV H5N7 A/waterfowl/GA/269452‐56/03	1	CU	+	+	−	−	−	−
cDNA of AIV AIV H5N9/chicken/QT35/98	1	CU	+	+	−	−	−	−
cDNA of AIV H7N2 AIV Duck/HK/47/76	1	HKU	+	−	−	+	−	−
cDNA of AIV H7N2/chicken PA/3979/97	1	PU	+	−	−	+	−	−
AIV H6N1 A/Duck/Guangxi/GXd‐5/2010	1	GVRI	+	−	+	−	−	−
AIV H6N1 A/duck/Guangxi/105/2011	1	GVRI	+	−	+	−	−	−
AIV H6N2 A/goose/Guangxi/105/2011	1	GVRI	+	−	−	+	−	−
AIV H6N2 A/goose/Guangxi/115/2012	1	GVRI	+	−	−	+	−	−
AIV H6N2 A/duck/Guangxi/116/2012	1	GVRI	+	−	−	+	−	−
AIVH6N2A/chicken/Guangxi/121/2013	1	GVRI	+	−	−	+	−	−
AIV H6N2 A/duck/Guangxi/121/2012	1	GVRI	+	−	−	+	−	−
AIV H9N2 A/turtledove/Guangxi/49B6/2013	1	GVRI	+	−	−	+	−	−
AIV H9N2 A/chicken/Guangxi/NN2/2011	1	GVRI	+	−	−	+	−	−
AIV H9N2 A/chicken/Guangxi/NN1/2011	1	GVRI	+	−	−	+	−	−
AIV H9N2 A/chicken/Guangxi/111C8/2012	1	GVRI	+	−	−	+	−	−
AIV H9N2 A/chicken/Guangxi/116C4/2012	1	GVRI	+	−	−	+	−	−
AIV H9N2 A/pheasant/Guangxi/49B2/2013	1	GVRI	+	−	−	+	−	−
AIV H9N2 A/sparrow/Guangxi/3 5B15/2013	1	GVRI	+	−	−	+	−	−
AIV H9N2 A/dove/Guangxi/31B6/2013	1	GVRI	+	−	−	+	−	−
AIV H9N2 A/chicken/Guangxi/141C10/2013	1	GVRI	+	−	−	+	−	−
AIV H9N2 A/chicken/Guangxi/CX/2013	1	GVRI	+	−	−	+	−	−
AIV H3N2 A/Chicken/Guangxi/015C10/2009	1	GVRI	+	−	−	+	−	−
AIV H3N2 A/Duck/Guangxi/015D2/2009	1	GVRI	+	−	−	+	−	−
AIV H3N6 A/pigeon/Guangxi/020P/2009	1	GVRI	+	−	−	−	+	−
AIV H3N6 A/Duck/Guangxi/175D12/2014	1	GVRI	+	−	−	−	+	−
AIV H6N6 A/duck/Guangxi/058/2010	1	GVRI	+	−	−	−	+	−
AIV H6N6 A/chicken/Guangxi/129/2013	1	GVRI	+	−	−	−	+	−
AIV H6N6 A/duck/Guangxi/131/2013	1	GVRI	+	−	−	−	+	−
AIV H6N6 A/pigeon/Guangxi/161/2014	1	GVRI	+	−	−	−	+	−
AIV H6N6 A/Duck/Guangxi/GXd‐7 /2011	1	GVRI	+	−	−	−	+	−
AIV H6N8 A/Duck/Guaiigxi/GXd‐6/2010	1	GVRI	+	−	−	−	−	+
AIV H6N8 A/duck/Guangxi/113/2012	1	GVRI	+	−	−	−	−	+
AIV H3N8 A/goose/Guangxi/020G/2009	1	GVRI	+	−	−	−	−	+
AIV H1N3 Duck/HK/717/79‐d1	1	HKU	+	−	−	−	−	−
AIVH1N1 Human/NJ/8/76	1	HKU	+	−	+	−	−	−
AIV H2N3 Duck/HK/77/76	1	HKU	+	−	−	−	−	−
AIV H3N6 AIV Duck/HK/526/79/2B	1	HKU	+	−	−	−	+	−
AIV H4N5 Duck/HK/668/79	1	HKU	+	−	−	−	−	−
AIV H6N8 Duck/HK/531/79	1	HKU	+	−	−	−	−	+
AIV H8N4 AIV Turkey/ont/6118/68	1	HKU	+	−	−	−	−	−
AIV H10N3 Duck/HK/876/80	1	HKU	+	−	−	−	−	−
AIVH11N3 Duck/HK/661/79	1	HKU	+	−	−	−	−	−
AIV H12N5 Duck/HK/862/80	1	HKU	+	−	−	−	−	−
AIV H13N5 AIV Gull/MD/704/77	1	HKU	+	−	−	−	−	−
AIV H13N6 A/Gull/Maryland/704/1977	1	PU	+	−	−	−	+	−
AIV H14N5 A/Mallard duck/Astrakhan/263/1982	1	PU	+	−	−	−	−	−
AIV H15N9	1	PU	+	−	−	−	−	−
A/wedge‐tailed shearwater/Western Australia/2576/1979								
AIV H16N3 A/shorebird/Delaware/168/06	1	PU	+	−	−	−	−	−
IAV H1N1 A/ Guangxi/1415/15	1	GCDC	−	−	−	−	−	−
IAV H3N2 A/ Guangxi/1632/15	1	GCDC	−	−	−	−	−	−
B/Guangxi/1470/15	1	GCDC	−	−	−	−	−	−
Other pathogens								
NDV Lasota	1	GVRI	−	−	−	−	−	−
IBV Massachussetts 41	1	GVRI	−	−	−	−	−	−
ILTV (AV1231)	1	GVRI	−	−	−	−	−	−
MGS6	1	GVRI	−	−	−	−	−	−
MSK1415	1	GVRI	−	−	−	−	−	−
HPGAV269	1	GVRI	−	−	−	−	−	−
Avian reovirus (S1133)	1	GVRI	−	−	−	−	−	−

HVRI, Harbin Veterinary Research Institute, China; HKU, The University of HongKong, China; GVRI, Guangxi Veterinary Research Institute, China; CIVDC, China Institute of Veterinary Drugs Control, China; PU, University of Pennsylvania, USA; GCDC, Guangxi Center for Disease Control; CU= University of Connecticut, USA.

### Primer design and plasmid preparation

Six pairs of gene‐specific primers were designed based on sequence information obtained from Influenza Sequence Database (http://www.flu.lanl.gov). The designed primers were analysed and filtered using the Premier 5.0 (Primer, Montreal, Canada), NCBI Primer Blast (NCBI, Bethesda, MD, USA) and Oligo 7.0 (Biolytic, Fremont, CA, USA) tool. Each gene‐specific primer was fused at the 5′ end to a universal sequence to generate six pairs of chimeric primers, and one pair of universal primers was used for RT‐PCR (Table 2). The AIV universal primers were designed to correspond to a highly conserved region of the matrix (M) gene, and the H5 primers were designed in a specific region of the HA gene segment for the H5 serotype. The other four pairs of primers were designed to correspond to the specific region of an NA gene segment for each of the NA types: N1, N2, N6 and N8. Primer synthesis and HPLC purification was performed by Invitrogen (Guangzhou, China).

**Table 2:** Sequences of primers used for GeXP assay

Primer	Forward primer (5→3)*	Reverse primer (5→3)*	Size (bp)
M	AGGTGACACTATAGAATATCTTGCACTTGAYATTGTGGATTC**	GTACGACTCACTATAGGGAACAAAATGACCATCGTCAACATCC**	211
H5	AGGTGACACTATAGAATAGGAAAGTGTAAGAAACGGAACGTA**	GTACGACTCACTATAGGGACACATCCATAAAGAYAGACCAGC**	223
N1	AGGTGACACTATAGAATA CTGTAATGACTGAYGGACCAAGTA**	GTACGACTCACTATAGGGACAGGAGCATTCCTCATAGTGGTAA**	162
N2	AGGTGACACTATAGAATAATGTTATCARTTTGCACTTGGGCAG**	GTACGACTCACTATAGGGA CATGCTATGCACACYTGTTTGGTTC**	188
N6	AGGTGACACTATAGAATACACTATAGATCCYGARATGATGACC**	GTACGACTCACTATAGGGAGGAGTCTTTGCTAATWGTCCTTCCA**	240
N8	AGGTGACACTATAGAATAATGTGTACCAGGCAAGGTTTGA**	GTACGACTCACTATAGGGATTTGCTGGTCCATCCGTCATTA**	280
Cy5‐labelled Tag‐F	AGGTGACACTATAGAATA **		
Tag‐R	GTACGACTCACTATAGGGA **		

Underlined oligonucleotides are universal sequences.

*Degenerated primer abbreviations are as follows: R, A/G; W, A/T; Y, C/T.

**Primer is in the PCR primer mix.

Plasmids harbouring genes from AIV (H5N1 AIV Re‐1, H5N2 AIV chicken/QT35/87, H3N6 AIV Duck/HK/526/79/2B, H6N8 AIV Duck/HK/531/79) were used to produce ssRNA via *in vitro* transcription using a RiboMAXTM large‐scale RNA production system‐T7 kit (Promega, Madison, WI, USA). The copy numbers of the ssRNAs for the target genes of AIV (M, H5, N1, N2, N6 and N8) were calculated according to previous methods.^20^, ^21^

### Set‐up of the multiplex PCR for the GeXP analyser

The reaction system was created using a total volume of 25 μl containing 2·5 μl of 10× PCR buffer, 2·5 μl of MgCl_2_ (25 μm), 1·25 μl of mixed primers (containing 20–100 nmol/l of 6 pairs of gene‐specific chimeric primers), 1·25 μl of universal primers (100 nmol/l), 1·2 μl of JumpStart Taq DNA polymerase (2·5 U/μl) and 0·5 pg–0·5 ng of cDNA or DNA template. Nuclease‐free water was then added to the PCR to achieve a final volume of 25 μl. PCR was carried out using the GeXP system followed by three steps of amplification according to the temperature switch PCR (TSP) strategy:^22^ step 1, 95°C for 3 minutes, 10 cycles of 95°C for 30 seconds, 55°C for 30 seconds and 72°C for 30 seconds; step 2, 10 cycles of 95°C for 30 seconds, 65°C for 30 seconds and 72°C for 30 seconds; and step 3, 20 cycles of 95°C for 30 seconds, 53°C for 30 seconds and 72°C for 30 seconds. The details of the primers are shown in Table 2, and all were purchased from Invitrogen. PCR product separation and detection were performed by capillary electrophoresis using the GenomeLab GeXP genetic analysis system (Beckman Coulter) following previously described protocols.^23^ After the amplified fragments were separated, the peaks were initially analysed using the fragment analysis module of the gexp system software (Beckman Coulter, Brea, CA, USA) and matched to the appropriate genes. The peak height for each gene is illustrated in an electropherogram.

### Single primer test for specificity of the GeXP multiplex assay

The assay specificities of all the targets were individually tested with pre‐mixed cDNA/DNA in a multiplex PCR assay after optimisation. Other conventional chicken viruses, including all subtypes (except the H5 subtype, including HA (1–16) and NA (1–9); the HA and NA genes of the reference strains were confirmed by sequencing) of avian influenza virus (AIV), Newcastle disease virus (NDV), infectious bronchitis virus (IBV), avian reovirus (ARV), infectious laryngotracheitis virus (ILTV), seasonal influenza A H1N1, H3N2 and influenza B viruses and nuclease‐free water, were used as negative controls.

### Evaluation of the sensitivity of the GeXP multiplex assay

The sensitivity of the multiplex assay using the GeXP analyser was evaluated using a previously described method.^23^ The concentrated products for each target gene were diluted to final concentrations ranging from 10^5^ copies to 1 copy per microlitre and then individually subjected to the multiplex assay. The concentrations of specific primers were optimised according to the amplification efficiency of the assay using a single template. The sensitivity of the optimised multiplex GeXP PCR assay was re‐evaluated three times on three different days using 6 pre‐mixed RNA templates ranging from 10^5^ copies to 1 copy per microlitre.

### Interference assay

Because high quantities of different templates could alter the efficiency of multiplex PCR amplification, different amounts of template (10^2^ to 10^6^ copies) were selected at random, mixed and tested in the multiplex PCR assay. The results were then compared with those of the single template multiplex PCR assay.

### Application to clinical specimens

A total of 180 cloacal swabs were collected from poultry at live bird markets (LBMs), and the swab samples were injected into 9‐ to 11‐day‐old embryonated specific pathogen‐free (SPF) chicken eggs, as previously described.^24^ At 48–96 hours, all allantoid fluids were recovered incubation for virus detection and titration, as described.^25^ The 180 clinical specimens were randomly divided into five groups and tested by RRT‐PCR, GeXP multiplex PCR and virus isolation, respectively (Table 3), and the HA and NA genes of positive samples were sequenced for demonstration of RT‐PCR compliance using previously reported primers.^26^

**Table 3:** Comparative detection of cloacal swab samples using virus isolation, RRT‐PCR and GeXP

Sample number	Virus isolate	Samples negative for avian influenza virus		Samples positive for avian influenza virus						
		RRT‐PCR	GeXP assay	RRT‐PCR	GeXP assay					
					M	H5	N1	N2	N6	N8
1–30	H5N1	29/30	29/30	1/30	1/30	1/30	1/30	0/30	0/30	0/30
31–60	H5N2	29/30	28/30	1/30	2/30	2/30	0/30	2/30	0/30	0/30
61–102	H9N2	39/42	38/42	3/42	4/42	0/42	0/42	4/42	0/42	0/42
103–137	H6N2	34/35	34/35	1/35	1/35	0/35	0/35	1/35	0/35	0/35
138–180	H6N6	41/43	41/43	2/43	2/43	0/43	0/43	0/43	2/43	0/43

## Results

### Specificity of the GeXP multiplex PCR assay

**Figure 1:**
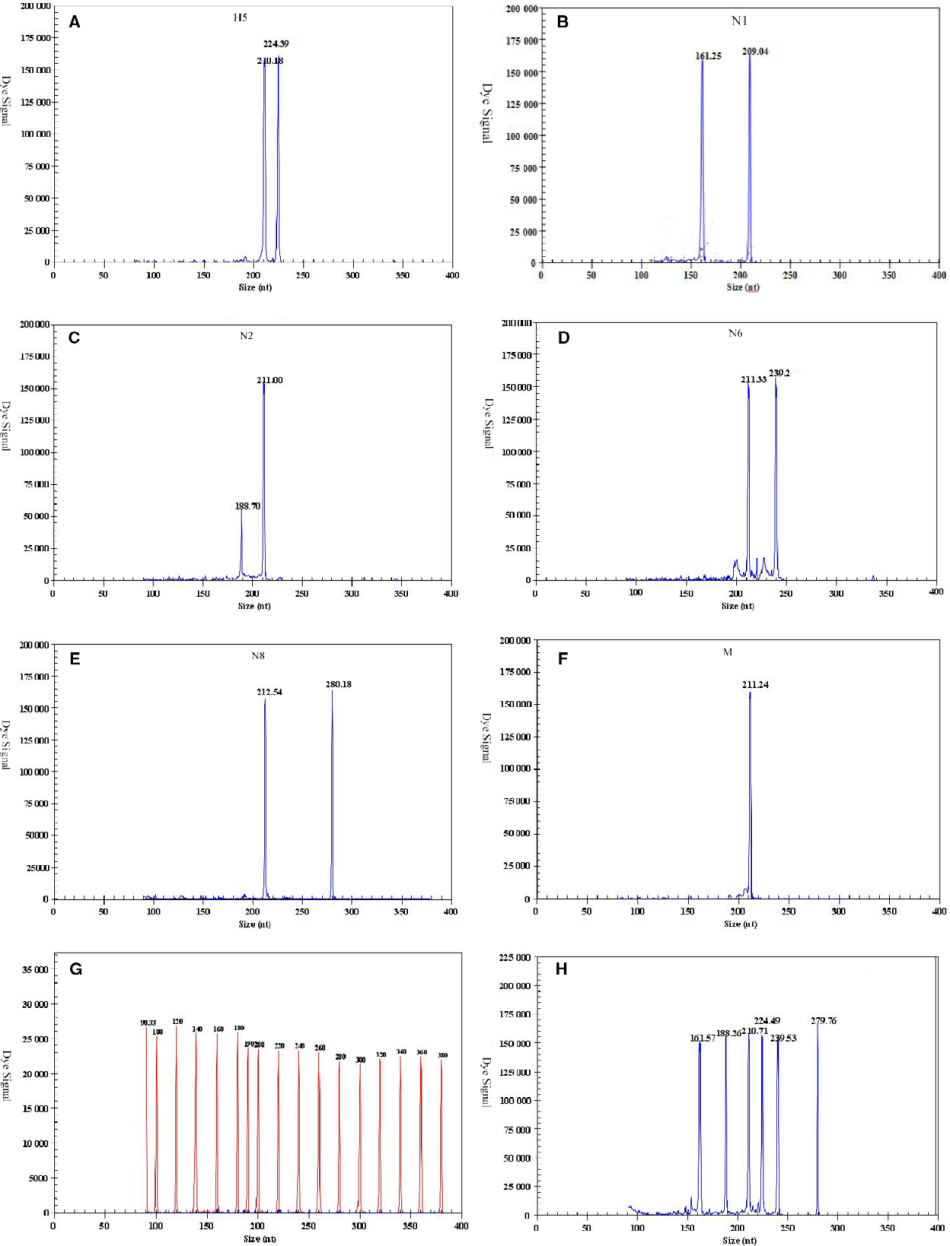
Specificity of the multiplex RT‐PCR assay. Cy5‐labeled PCR products were separated via GeXP capillary electrophoresis and detected by fluorescence spectrophotometry, given as dye signals in arbitrary units on the y axis. Each peak was identified by comparing the expected to the actual PCR product size on the x axis. Panels (A‐F, H) show the results of amplification of target genes H5, N1, N2, N6, N8, M, and all, respectively. Nuclease‐free water was used as the negative control (G). the red peaks indicate the DNA size standard.

cDNA samples from four different NA subtypes of influenza A H5 virus (H5N1, H5N2, H5N6 and H5N8) were individually used as template to evaluate the specificity of each pair of gene‐specific primers. In mono‐PCR GeXP assays, AIV universal primers were able to amplify the target M gene of all AIV serotypes, although each pair of specific primers generated only the corresponding targeted gene, without cross‐amplification (Table 1). The amplicon sizes for the viruses were as follows: AIV‐H5, 223–226 bp; AIV‐N1, 160–163 bp; AIV‐N2, 188–191 bp; AIV‐N6, 240–243 bp; AIV‐N8, 280–283 bp; and AIV M gene, 210–213 bp (Table 2). These six targeted genes were detected via the multiplex GeXP PCR assay, and specific amplification peaks were observed (Figure 1). For example, specific amplification peaks of the AIV M gene were observed for H1N1, H2N3, H3N6, H4N5, H5N2, H5N3, H5N7, H6N8, H7N2, H8N4, H9N2, H10N3, H11N3, H12N5, H13N6, H14 N5, H15N9 and H16N3.

### Sensitivity of the GeXP multiplex PCR assay

**Figure 2:**
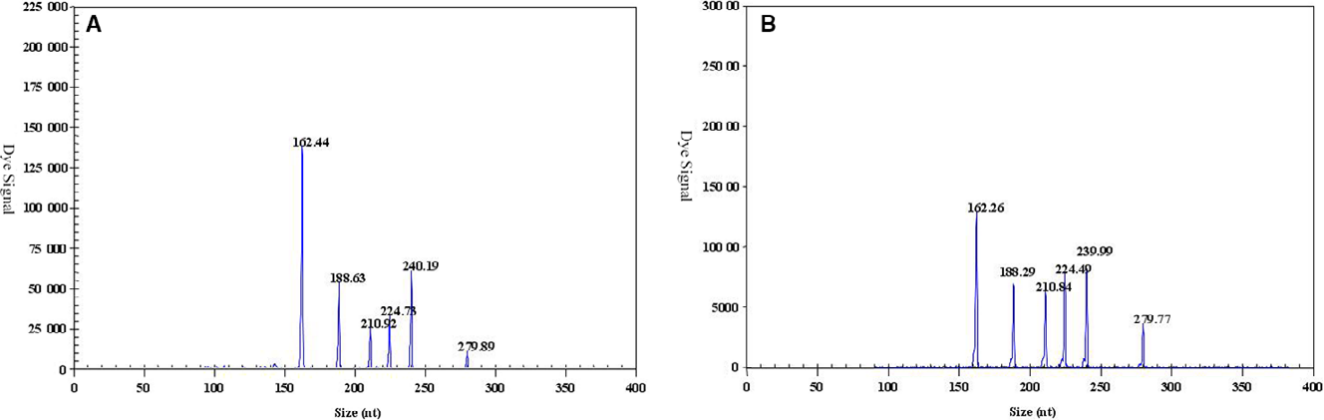
Sensitivity of GeXP detection of 6 premixed RNA templates with multiplex primers. All the 6 target genes could be detected at 10^3^ copies/μl (A) and 10^2^ copies/μl (B).

When using ssRNA *in vitro* transcription individually, the GeXP multiplex PCR assay detected as little as 10^2^ copies/μl of AIV type A, AIV‐H5, AIV‐N1, AIV‐N2, AIV‐N6 and AIV‐N8 (Figure 2, all electropherograms are not shown). The reactions were repeated three times for each template concentration on different days, and similar results were obtained.

### Artificial mixture and interference assay

Two specific amplification peaks were observed when two different templates (one template with 10^2^ copies and another with 10^6^ copies) were tested by the GeXP multiplex PCR assay, and the peak value of the single template was similar to that of the mixed template. For example, two specific amplification peaks were observed when two different templates (AIV‐H5N1 at 10^2^ copies and H5N2 at 10^6^ copies) were tested, and the peak values for AIV‐H5N1 and H5N2 were the same, regardless of whether a single (AIV‐H5N1 or H5N2) or a mixed (AIV‐H5N1 + H5N2) template was utilised (electropherograms not shown). The results of these experiments demonstrate that this interference had a minimal effect on the detection of mixed infection.

### Detection of clinical samples by the GeXP multiplex PCR assay

A total of 180 random cloacal swab samples were collected from poultry at various LBMs during May 2015; the samples were assayed using optimised GeXP multiplex PCR (Tables 3) and confirmed by DNA sequencing of the HA and NA genes. Virus isolation revealed ten different genotypes among the clinical samples, and three positive samples of H5‐subtype AIVs were isolated among 60 cloacal swab samples. Of the ten positive samples according to the GeXP PCR assay, one sample was negative by RRT‐PCR but positive when retested by virus isolation. Moreover, the HA and NA genes of the positive samples were sequenced, showing complete agreement for 100% of the GeXP PCR assay results (Table 3). We also found some nucleotide variation, which resulted in the failure of the real‐time PCR detection of an H9N2 isolate.

## Discussion

Although the vaccination strategy against H5N1 influenza has been effective at reducing the incidence of H5N1 infection in poultry during the past decade, recent studies have highlighted the continued presence of H5N1, H5N2, H5N6 and H5N8, posing a threat to both poultry and human health.^27^, ^28^ Outbreaks of various H5 HPAIV NA subtypes are characterised by their sudden occurrence, dissemination speed, similar clinical symptoms and highly infectious nature with common characteristics. As such factors are unfavourable for the implementation of rational measures to prevent and control disease, the rapid and accurate identification of different NA subtypes circulating in different avian species within a geographical region can provide important epidemiological information and is crucial for the selection of appropriate control and eradication strategies.

Although HI and NI tests have long been used as the Office International Des Epizooties (OIE) standard for AIV subtyping, these methods often have low levels of specificity and sensitivity; in addition, standardisation and reference antisera are difficult to obtain.^29^ Accordingly, laboratory diagnosis of AIV, including conventional and real‐time RT‐PCR protocols, has been recently used for AIV detection and subtyping.^9^, ^30^, ^31^, ^32^ Indeed, multiplex PCR and multiplex fluorescence real‐time quantitative PCR techniques are widely used for the detection and subtyping of mixed infections with multiple pathogens.^33^, ^34^ However, conventional multiple PCR is vulnerable to variations in template and primer concentration as well as concentrations of reagents that interfere with each other during amplification; such factors will cause different PCR efficiencies, decreasing the sensitivity of the results.

In this study, we successfully established a GeXP method that can simultaneously detect and identify the H5N1, H5N2, H5N6 and H5N8 subtypes of AIV in a single reaction within 4 hours; this GeXP method utilises six different genes primers for M, H5, N1, N2, N6 and N8. Additionally, the specificity of the test was examined using different HA (1–16) and NA (1–9) subtypes of avian influenza virus and nucleic acid of seasonal influenza virus and influenza B virus infecting humans (Table 1). The failure of the M gene primer set in detecting human H1N1 and H3N2 is because the primer set is based on GenBank data of avian influenza virus, with variation in a few nucleotides between human H1N1 and H3N2. We also found no cross‐reaction between different pair primers, with specificity for detecting AIV H5, N1, N2, N6 and N8 subtypes and a sensitivity of 10^2^ copies/μl. When 180 specimens were analysed, the results for the GeXP PCR assay and the reference method (Virus isolation and sequencing) were in complete agreement for 100% of the specimens, as shown in Table 3. However, one H9N2 isolate was not detected by RRT‐PCR using the N2 primer set (Table 3). The comparison of the results for NA sequences revealed that the new H9N2 isolates exhibited variation in the target gene of the N2 primer designed for RRT‐PCR. Considering that the genetic diversity of AIVs in the natural ecosystem may be higher than the current understanding, it may be necessary to develop primers for all subtypes.

Two distinct advantages of the GeXP assay compared with other nucleic acid‐based tests are the reduced time required and cost‐effectiveness. The cost of the multiplex GeXP assay for simultaneous detection of the four different NA subtypes of H5 AIVs is approximately $6 per test, including primers representing the HA and NA subtypes, when the entire reaction is performed in a single tube. Utilising two 96‐well plates at the same time in the GeXP analyser will further increase the throughput. This method will save much time and cost compared with classical methods of AIV HA and NA subtyping in surveillance programmes.

## Author contributions

Zhi‐xun Xie designed and coordinated the study. Meng Li designed the primers, optimised the conditions of the GeXP assay and finalised the analysis. All other authors offered much help in the supplement of different clinical samples, shared previous experimental data and approved the final version of the manuscript.

## Conflicts of interest

We declare that we have no conflicts of interest.

## References

[1] Zhu X , Yu W , McBride R *et al* Hemagglutinin homologe from H17N10 bat influenza virus exhibits divergent receptor‐binding and pH‐dependent fusion activities. Proc Natl Acad Sci USA 2013; 110:1458–1463. 23297216 10.1073/pnas.1218509110 PMC3557073

[2] Tong S , Zhu X , Li Y *et al* New world bats harbor diverse influenza A viruses. PLoS Pathog 2013; 9:e1003657. 24130481 10.1371/journal.ppat.1003657 PMC3794996

[3] Swayne DE , Suarez DL . Highly pathogenic avian influenza. Rev Sci Tech 2000; 19:463–482. 10935274 10.20506/rst.19.2.1230

[4] Sun H , Liu J . Epidemicity and pathogenicity of avian influenza A H5N1 virus. Chin Bull Life Sci 2015; 27:525–530.

[5] Jeong J , Kang HM , Lee EK *et al* Highly pathogenic avian influenza virus (H5N8) in domestic poultry and its relationship with migratory birds in South Korea during 2014. Vet Microbiol 2014; 173:249–257. 25192767 10.1016/j.vetmic.2014.08.002

[6] OIE . Avian influenza portal. 2015 Available at http://www.oie.int/animal-health-in-the-world/update-on-avian-influenza/2015/ (Accessed 10 August 2015).

[7] Chen H . H5N1 avian influenza in China. Sci China Ser C Life Sci 2009; 52:419–427. 19471864 10.1007/s11427-009-0068-6

[8] Xie Z , Pang YS , Liu J *et al* A multiplex RT‐PCR for detection of type A influenza virus and differentiation of avian H5, H7, and H9 hemagglutinin subtypes. Mol Cell Probes 2006; 20:245–249. 16542820 10.1016/j.mcp.2006.01.003

[9] Tsukamoto K , Panei CJ , Shishido M *et al* SYBR green‐based real‐time reverse transcription‐PCR for typing and subtyping of all hemagglutinin and neuraminidase genes of avian influenza viruses and comparison to standard serological subtyping tests. J Clin Microbiol 2012; 50:37–45. 22031706 10.1128/JCM.01195-11 PMC3256701

[10] Heine HG , Foord AJ , Wang J *et al* Detection of highly pathogenic Zoonotic Influenza Virus H5N6 chain reaction. Virol J 2015; 8:12–18. 10.1186/s12985-015-0250-3 PMC4328077 25889293

[11] Liu Y , Xu ZQ , Li JS *et al* A novel method for multiplex detection of gastroenteritis‐associated viruses. Bing Du Xue Bao 2011; 27:288–293. 21774256

[12] Hu X , Zhang Y , Zhou X *et al* Simultaneously typing nine serotypes of enteroviruses associated with hand, foot, and mouth disease by a GeXP analyzer‐based multiplex reverse transcription‐PCR assay. J Clin Microbiol 2012; 50:288–293. 22116146 10.1128/JCM.05828-11 PMC3264198

[13] Qin M , Wang DY , Huang F *et al* Detection of pandemic influenza AH1N1 virus by multiplex reverse transcription‐PCR with a GeXP analyser. J Virol Methods 2010; 168:255–258. 20452377 10.1016/j.jviromet.2010.04.031

[14] Li J , Qi S , Zhang C *et al* A two‐tube multiplex reverse transcription PCR assay for simultaneous detection of sixteen human respiratory virus types/subtypes. Biomed Res Int 2013; 2013:327620. 23984344 10.1155/2013/327620 PMC3747601

[15] Liu Y , Xu Z , Zhang Q *et al* Simultaneous detection of seven enteric viruses associated with acute gastroenteritis by a multiplexed Luminex‐based assay. J Clin Microbiol 2012; 50:2384–2389. 22518865 10.1128/JCM.06790-11 PMC3405628

[16] Yang M , Luo L , Nie K *et al* Genotyping of 11 human papillomaviruses by multiplex PCR with a GeXP analyzer. J Med Virol 2012; 84:957–963. 22499019 10.1002/jmv.23275

[17] Xie Z , Luo S , Xie L *et al* Simultaneous typing of nine avian respiratory pathogens using a novel GeXP analyzer‐based multiplex PCR assay. J Virol Methods 2014; 207:188–195. 25025815 10.1016/j.jviromet.2014.07.007

[18] Zhang M , Xie Z , Xie L *et al* Simultaneous detection of eight swine reproductive and respiratory pathogens using a novel GeXP analyser‐based multiplex PCR assay. J Virol Methods 2015; 224:9–15. 26259690 10.1016/j.jviromet.2015.08.001

[19] Zhang YF , Xie ZX , Xie LJ *et al* GeXP analyzer‐based multiplex reverse‐transcription PCR assay for the simultaneous detection anddifferentiation of eleven duck viruses. BMC Microbiol 2015; 15:247. 26518004 10.1186/s12866-015-0590-6 PMC4628294

[20] Xie Z , Xie L , Pang Y *et al* Development of a real‐time multiplex PCR assay for detection of viral pathogens of penaeid shrimp. Arch Virol 2008; 153:2245–2251. 19018451 10.1007/s00705-008-0253-0

[21] Xie Z , Xie L , Fan Q *et al* A duplex quantitative real‐time PCR assay for the detection of Haplosporidium and Perkinsus species in shellfish. Parasitol Res 2013; 112:1597–1606. 23371501 10.1007/s00436-013-3315-5

[22] Tabone T , Mather DE , Hayden MJ . Temperature switch PCR (TSP): robust assay design for reliable amplification and genotyping of SNPs. BMC Genom 2009; 510:580. 10.1186/1471-2164-10-580 PMC2795770 19958555

[23] Rai AJ , Kamath RM , Gerald W , Fleisher M . Analytical validation of the GeXP analyzer and design of a work flow for cancer‐bio marker discovery using multiplexed gene‐expression profiling. Anal Bioanal Chem 2009; 393:1505–1511. 18958454 10.1007/s00216-008-2436-7

[24] Peng Y , Xie Z , Liu J *et al* Epidemiological surveillance of low patho‐genic avian influenza virus (LPAIV) from poultry in Guangxi Province, Southern China. PLoS One 2013; 8:e77132. 24204754 10.1371/journal.pone.0077132 PMC3813733

[25] Xie Z , Dong J , Tang X *et al* Sequence and phylogenetic analysis of three isolates of avian influenza H9N2 from chickens in Southern China. Sch Res Exch 2008; 2008a:1–7.

[26] Hoffmann E , Stech J , Guan Y , Webster RG , Perez DR . Universal primer set for the full‐length amplification of all influenza A viruses. Arch Virol 2001; 146:2275–2289. 11811679 10.1007/s007050170002

[27] Chen H . Avian influenza vaccination: the experience in China. Rev Sci Tech 2009; 28:267–274. 19618631 10.20506/rst.28.1.1860

[28] Wang M , Di B , Zhou D *et al* Food markets with live birds as source of avian influenza. Emerg Infect Dis 2006; 12:1773–1775. 17283635 10.3201/eid1211.060675 PMC3372357

[29] Fouchier RAM , Munster V , Wallensten A *et al* Characterization of a novel influenza A virus hemagglutinin subtype (H16) obtained from black‐headed gulls. J Virol 2005; 79:2814–2822. 15709000 10.1128/JVI.79.5.2814-2822.2005 PMC548452

[30] Huang Y , Khan MI , Măndoiu I . Neuraminidase subtyping of avian influenza viruses with primerhunter‐designed primers and quadruplicate primer pools. PLoS One 2013; 8:e81842. 24312367 10.1371/journal.pone.0081842 PMC3843705

[31] Qiu B , Liu W , Peng D , Hu S , Tang Y , Liu X . A reverse transcription‐PCR for subtyping of the neuraminidase of avian influenza viruses. J Virol Methods 2009; 155:193–198. 18984006 10.1016/j.jviromet.2008.10.001

[32] Elizalde M , Agüero M , Buitrago D *et al* Rapid molecular haemagglutinin subtyping of avian influenza isolates by specific real‐time RT‐PCR tests. J Virol Methods 2014; 196:71–81. 24184949 10.1016/j.jviromet.2013.10.031

[33] Pang Y , Wang H , Girshick T , Xie Z , Khan MI . Development and application of a multiplex polymerase chain reaction for avian respiratory agents. Avian Dis 2002; 46:691–699. 12243534 10.1637/0005-2086(2002)046[0691:DAAOAM]2.0.CO;2

[34] Kuriakose T , Hilt DA , Jackwood MW . Detection of avian influenza viruses and differentiation of H5, H7, N1, and N2 subtypes using a multiplex microsphere assay. Avian Dis 2012; 56:90–96. 22545533 10.1637/9828-060211-Reg.1

